# Bayesian Statistics to Reanalyze Data From the STAAMP Trial

**DOI:** 10.1001/jamanetworkopen.2026.2112

**Published:** 2026-03-17

**Authors:** Danielle S. Gruen, James Matuk, Francis X. Guyette, Joshua B. Brown, Christine M. Leeper, Brian J. Eastridge, Raminder Nirula, Gary A. Vercruysse, Terence O’Keeffe, Bellal Joseph, Stephen Wisniewski, Matthew D. Neal, Jason L. Sperry

**Affiliations:** 1 Department of Surgery, University of Pittsburgh, Pittsburgh, Pennsylvania; 2 Department of Epidemiology, University of Pittsburgh, Pittsburgh, Pennsylvania; 3 Department of Emergency Medicine, University of Pittsburgh, Pittsburgh, Pennsylvania; 4 Department of Surgery, The University of Texas Health Science Center at San Antonio; 5 Department of Surgery, University of Utah Health, Salt Lake City; 6 Department of Surgery, University of Michigan, Ann Arbor; 7 Department of Surgery, Augusta University, Augusta, Georgia; 8 Department of Surgery, Banner-University Medical Center Tucson, Tucson, Arizona

## Abstract

**Importance:**

Tranexamic acid (TXA) is associated with improved survival following trauma in prior prehospital trials, shaping clinical practice. The Study of Tranexamic Acid During Air and Ground Medical Prehospital Transport (STAAMP) trial did not find a difference in mortality between TXA and placebo. A bayesian approach that incorporates prior clinical evidence may better characterize the impact of TXA.

**Objective:**

To evaluate the probability of mortality benefit associated with prehospital TXA in trauma.

**Design, Setting, and Participants:**

This quality improvement study used a post hoc bayesian analysis of data from the STAAMP trial, a prospective, multicenter, double-masked, placebo-controlled, phase 3 randomized clinical trial conducted at level I trauma centers in the US from May 1, 2015, to October 31, 2019. Patients at risk for hemorrhage within approximately 2 hours of injury were randomized to receive prehospital TXA or placebo. The data analysis was performed between January 1, 2024, and December 31, 2025.

**Main Outcomes and Measures:**

The primary outcome was 30-day mortality assessed using frequentist statistics. Bayesian hierarchical logistic regression models were built to estimate the posterior probability of mortality associated with TXA. The prior distributions were informed by Clinical Randomization of an Antifibrinolytic in Significant Hemorrhage 2 (CRASH-2) and Prehospital Antifibrinolytics for Traumatic Coagulopathy and Hemorrhage (PATCH-Trauma) trial data, and the influence of prior selection, sample size, and varying risk thresholds was also evaluated.

**Results:**

Of 903 STAAMP patients analyzed (median [IQR] age, 39 [26-55] years; 668 male [74.0%]), 447 received TXA and 456 received placebo. The majority of patients (n = 760 [84.2%]) sustained blunt injuries, with a median injury severity score of 12 (IQR, 5-22). In the frequentist analysis, the 30-day mortality rates were 8.1% and 9.9% for the TXA and placebo groups, respectively (hazard ratio, 0.81; 95% CI, 0.59-1.11). Using bayesian models, the estimated posterior risk ratios were 0.91 (95% credible interval [CrI], 0.85-0.97) with a CRASH-2 prior, 0.80 (95% CrI, 0.65-0.97) with a PATCH-Trauma prior, and 0.82 (95% CrI, 0.52-1.23) under a noninformative prior. The results were robust across confounders, site clustering, and alternative priors. The posterior probability that TXA reduced mortality ranged from 84% to 99%.

**Conclusions and Relevance:**

This quality improvement study using a post hoc bayesian reanalysis of the STAAMP trial suggested a high probability that prehospital TXA would improve survival. Bayesian methods may offer refined inference and support clinical decision-making in prehospital trauma care.

## Key Points

**Question:**

What is the probability that prehospital tranexamic acid (TXA) is associated with reduced 30-day mortality in patients with trauma at risk of hemorrhage when analyzed with a bayesian approach?

**Findings:**

In this quality improvement study with a post hoc bayesian analysis of the STAAMP trial in 903 patients, the posterior probability that TXA was associated with a 30-day mortality benefit ranged from 84% to 99%, including under a noninformative prior.

**Meaning:**

These findings suggest that prehospital TXA is associated with a high probability of improved survival, and bayesian methods may enhance inference by quantifying the probability of benefit and incorporating prior clinical evidence.

## Teaser

This quality improvement study reanalyzes data from the Study of Tranexamic Acid During Air and Ground Medical Prehospital Transport (STAAMP) trial using bayesian methods to evaluate the posterior probability that prehospital tranexamic acid is associated with reduced 30-day mortality in patients with trauma at risk of hemorrhage.

## Introduction

Severe hemorrhage from traumatic injury is a leading cause of morbidity and mortality in military and civilian populations.^1,2^ Interventions aimed at early hemorrhage control improve survival following trauma.^2,3,4,5^ Tranexamic acid (TXA), which inhibits fibrinolysis and stabilizes clot formation, has been used to reduce bleeding in surgical settings. The landmark Clinical Randomization of an Antifibrinolytic in Significant Hemorrhage 2 (CRASH-2) trial demonstrated lower mortality when TXA was administered to bleeding patients close to the time of traumatic injury. The CRASH-2 trial randomized more than 20 000 injured patients with positive results.^6^ Large, prospective, multicenter, masked, placebo-controlled, randomized clinical trials are the criterion standard for advancing clinical practice. Such trials are difficult to deploy in prehospital trauma research due to inherent challenges, such as the time-sensitive nature and acuity of disease and logistical, ethical, and funding limitations.^7^

Smaller subsequent prehospital TXA trials have generated nuanced results. The Study of Tranexamic Acid During Air and Ground Medical Prehospital Transport (STAAMP) trial demonstrated improved survival in specific subgroups of patients who received prehospital TXA but no difference in the overall cohort.^8,9,10^ It was hypothesized that early TXA administration may mitigate the endotheliopathy of trauma, but the trial was underpowered to detect an outcome difference in the overall cohort using frequentist methods.^8,11^ More recently, the Prehospital Antifibrinolytics for Traumatic Coagulopathy and Hemorrhage (PATCH-Trauma) trial supported a TXA-associated 28-day survival benefit.^12^ The true magnitude of TXA’s benefit in prehospital trauma care remains uncertain.

Bayesian methods offer an alternative framework for analyzing clinical trial data by incorporating prior evidence to refine effect estimates and improve inference. Traditional frequentist methods assess the probability of obtaining the observed result or something more extreme under the null hypothesis using an arbitrarily defined *P* value (eg, .05) and do not reveal a magnitude of the effect.^13^ Bayesian approaches estimate the probability that an intervention works or causes harm.^14,15^ Unlike frequentist approaches which rely solely on data from the study at hand, bayesian methods integrate prior knowledge, potentially increasing statistical power and generating more robust conclusions.^7,16,17^ Importantly, bayesian approaches can apply varying degrees of belief about prior evidence (ie, assuming skeptical, neutral, or optimistic or enthusiastic priors) to examine how prior assumptions shape the posterior distribution of the treatment effect.^18^ The value of this approach was recently illustrated by a post hoc bayesian analysis of the Pragmatic Randomized Optimal Platelet and Plasma Ratios trial, which quantified the mortality benefit for various transfusion strategies.^19,20^

Given the potential importance of TXA in prehospital trauma care, we sought to better quantify using a bayesian approach the probability that TXA reduces 30-day mortality in patients at risk for hemorrhage transported by air and ground ambulance. Our objectives were to understand the association between TXA and survival, evaluate the influence of prior selection, and compare our findings with results from frequentist analyses. We hypothesized that there is a survival benefit associated with prehospital TXA compared with standard care when analyzed using a bayesian approach.

## Methods

### Study Design and Patient Population

This quality improvement study is a post hoc bayesian analysis of the STAAMP trial, a prospective, multicenter, double-masked, placebo-controlled, phase 3 randomized clinical trial.^21^ The STAAMP trial was approved under the Emergency Exception From Informed Consent protocol from the Human Research Protection Office of the US Army Medical Research and Material Command. The trial was approved by the institutional review boards of each participating site. The study adhered to the Declaration of Helsinki,^22^ and followed the Consolidated Standards of Reporting Trials (CONSORT) reporting guideline. This post hoc bayesian analysis was conducted under the same ethical oversight and followed the Standards for Quality Improvement Reporting Excellence (SQUIRE) reporting guideline as appropriate.^23^ The full study protocol is publicly available.^21^

The STAAMP trial was conducted across multiple level 1 trauma centers in the US to evaluate the effectiveness of prehospital TXA administration in patients with trauma at risk of hemorrhage. We included patients at risk for hemorrhage based on prehospital hypotension (systolic blood pressure <90 mm Hg) or tachycardia (heart rate >110 beats per minute) before arrival to the hospital within an estimated 2 hours of injury from May 1, 2015, through October 31, 2019. Patients were randomized to receive 1 g of prehospital TXA or placebo (saline) infused for 10 minutes followed by an in-hospital phase. The primary outcome was 30-day all-cause mortality.^8^

### Statistical Analysis

#### Frequentist Analysis

The STAAMP trial data were analyzed using traditional frequentist methods at the primary study end point. A Cox proportional hazards model was used to estimate the hazard ratio and 95% CI for mortality associated with TXA administration. The primary study frequentist analysis was performed at the conclusion of the study from May 1, 2019, to October 31, 2020.

#### Bayesian Model Development and Prior Specification

We developed Bayesian hierarchical logistic regression models to estimate the posterior probability of mortality associated with TXA. We incorporated data from the CRASH-2 and PATCH-Trauma trials, defining our prior based on the relative risk reduction observed in these studies at the primary study end point (30-day or 28-day mortality, respectively). The outcome variable was modeled using a Bernoulli family distribution. Bayesian models and analysis were completed from January 1, 2024, to December 31, 2025.

To evaluate our assumptions and assess the level of influence of prior selection, we conducted sensitivity analyses assuming varying degrees of enthusiasm or skepticism for prior trial evidence. We derived our optimistic prior from the log-transformed risk ratio (RR) and SE observed in CRASH-2 (RR, 0.91; 95% CI, 0.85-0.97). We used a weakly informative, normally distributed prior for the intercept. We used a normal prior centered on the log RR from CRASH-2, the largest randomized clinical trial of TXA in trauma. We evaluated models with deflated prior precision (increasing SDs of 0.05 and 0.10 to simulate smaller CRASH-2 populations), a prior based on a separate study population with PATCH-Trauma data (RR, 0.79; 95% CI, 0.63-0.99), and a diffusely distributed noninformative (neutral) prior (assuming a normal distribution and SD of 1000). We also accounted for known confounding variables in this study population, including age, injury severity score, prehospital transfer status, and clustering by enrolling site with hierarchical modeling.

All models were developed using the brms package, version 2.22.0,^24,25,26^ and statistical analyses were performed using R, version 4.3.3 (R Foundation for Statistical Computing).^27^ Sample code is available in the eMethods in Supplement 1.

#### Model Evaluation

Posterior inference was enabled using Markov chain Monte Carlo methods. Models were analyzed with the R brms package using a No-U-turn sampler.^24,25,26^ We ran 4 randomly initialized Markov chains to sample from the target posterior distribution, each with 2000 iterations (1000 burn-in), for a total of 4000 draws. Convergence diagnostics, including Rhat statistics and trace plots, were evaluated to ensure model stability.

We analyzed the posterior distributions of our models to evaluate the probability of a survival benefit associated with TXA. Posterior distributions of treatment effect estimates were summarized using the mean posterior RR and corresponding 95% credible interval (CrI). Evidence for treatment benefit was assessed based on the probability of TXA reducing mortality (ie, the proportion of the posterior distribution with RR <1.0). To assess the strength of evidence and robustness across varying risk thresholds, we calculated the posterior probability that TXA reduced mortality below clinically meaningful RR cutoffs (eg, RR <1.0, RR <0.9, RR <0.8) and estimated Bayes factors (BFs) for each posterior computed as evidence ratios for 1-sided hypothesis tests based on posterior samples. The BFs were interpreted using the Jeffreys scale.^28,29^

## Results

A total of 903 patients (median [IQR] age, 39 [26-55] years; 235 female [26.0%] and 668 male [74.0%]) were included in the analysis, with 447 randomized to receive TXA and 456 to receive placebo. Data on race and ethnicity were collected and reported in the primary analysis^8^ and were not analyzed further in this study. Patients primarily sustained blunt injuries (760 [84.2%]) with a median injury severity score of 12 (IQR, 5-22), and 212 (23.6%) sustained a traumatic brain injury. A total of 127 patients (14.3%) were transferred from another facility (Table 1).

**Table 1.** Baseline Characteristics of the Study Cohort by Treatment Group

Characteristic	Participants, No. (%)		
	Overall (N = 903)	Placebo (n = 456)	TXA (n = 447)
Age, median (IQR), y	39 (26-55)	39 (26-56)	39 (26-53)
Sex			
Female	235 (26.0)	115 (25.2)	120 (26.8)
Male	668 (74.0)	341 (74.8)	327 (73.2)
Blunt injury	760 (84.2)	389 (85.3)	371 (83.0)
TBI	212 (23.6)	106 (23.3)	106 (23.8)
ISS, median (IQR)	12 (5-22)	11 (4-22)	13 (5-22)
Transfer status	127 (14.3)	61 (13.5)	66 (15.1)
30-d Mortality	81 (9.0)	45 (9.9)	36 (8.1)

Abbreviations: ISS, injury severity score; TBI, traumatic brain injury; TXA, tranexamic acid.

In a frequentist analysis of the STAAMP trial, the 30-day mortality rate was 8.1% in the TXA group vs 9.9% in the placebo group (absolute difference, −1.8 percentage points; 95% CI, −5.6 to 1.9 percentage points; *P* = .17). The hazard ratio for mortality associated with TXA was 0.81 (95% CI, 0.59-1.11; *P* = .18), which failed to reject the null hypothesis that there is no difference in survival between treatment groups.

**Figure 1.**
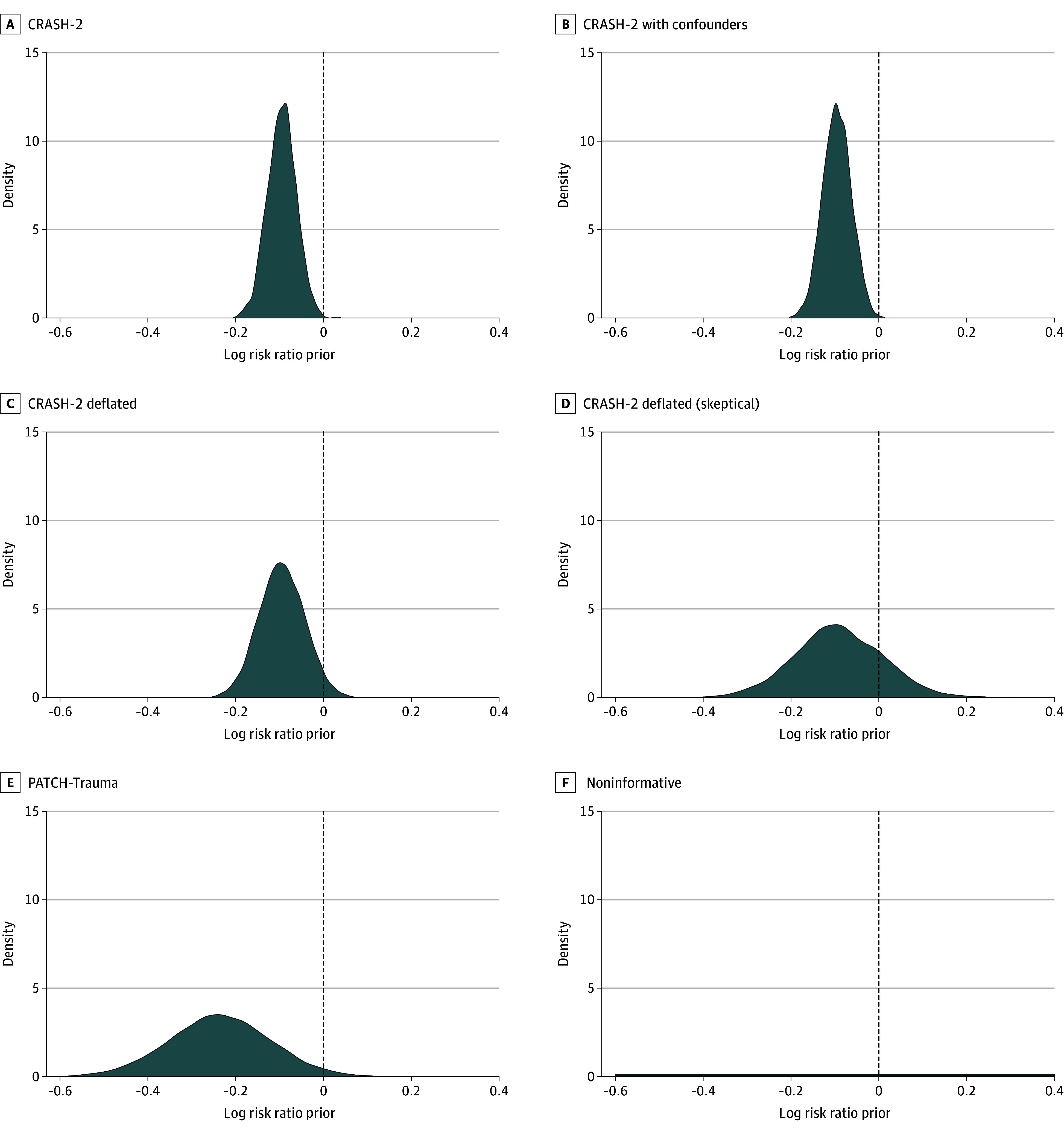
Prior Distribution Plots Showing Log Risk Ratio Prior Density by Model The area under the curve to the left of 0 (risk ratio <1) (dashed vertical line) indicates the posterior probability that tranexamic acid reduces mortality. CRASH-2 indicates Clinical Randomization of an Antifibrinolytic in Significant Hemorrhage 2; PATCH-Trauma, Prehospital Antifibrinolytics for Traumatic Coagulopathy and Hemorrhage.

Bayesian hierarchical logistic regression models were constructed using priors informed by the CRASH-2 and PATCH-Trauma trials and compared with a noninformative prior. Prior distributions ranged from more informative based on CRASH-2 and PATCH-Trauma to a more diffuse noninformative prior (Figure 1). As prior strength decreased, the distributions became wider, reflecting increasing uncertainty and more skeptical assumptions about the magnitude of TXA’s treatment effect.

**Figure 2.**
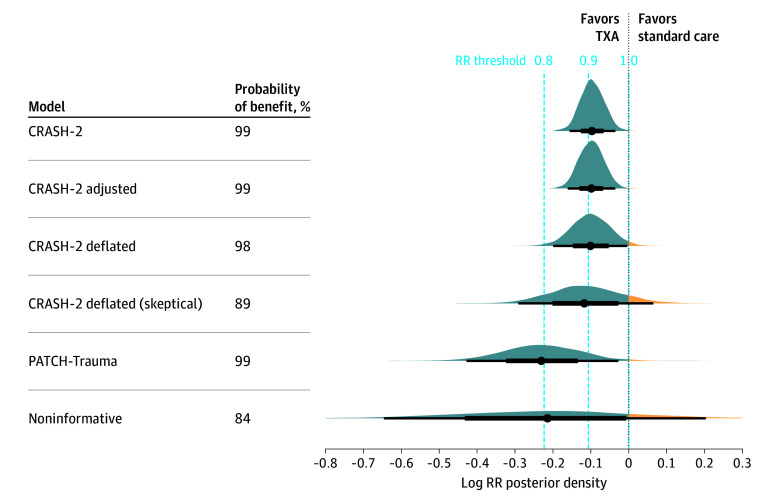
Posterior Distribution Plots Showing the Log Risk Ratio Posterior Density by Model Shaded curves indicate the posterior distributions of the log risk ratio for each bayesian model estimating the effect of tranexamic acid (TXA) on 30-day mortality. Vertical dashed lines represent key risk ratio (RR) thresholds. The area under the curve to the left of 0 (eg, log RR <0 or RR <1), represented by the blue shading, indicates the posterior probability that TXA would reduce mortality. The area under the curve to the right of 0 (log RR >0), represented by the orange shading, indicates the posterior probability that placebo is beneficial. The percentages labeled represent the posterior probability that the RR is less than 1, ie, the probability that TXA is beneficial in each model. Dots indicate point estimates; thick lines, the 50% credible intervals; thin lines, the 95% credible intervals. CRASH-2 indicates Clinical Randomization of an Antifibrinolytic in Significant Hemorrhage 2; PATCH-Trauma, Prehospital Antifibrinolytics for Traumatic Coagulopathy and Hemorrhage.

The posterior mean RRs for 30-day mortality were 0.91 (95% CrI, 0.85-0.97) for the CRASH-2 prior, 0.91 (95% CrI, 0.85-0.97) for the CRASH-2 prior with confounders, 0.91 (95% CrI, 0.82-1.00) for the deflated CRASH-2 prior, 0.90 (95% CrI, 0.75-1.07) for the most skeptical deflated CRASH-2 prior, 0.80 (95% CrI, 0.65-0.97) for the PATCH-Trauma prior, and 0.82 (95% CrI, 0.52-1.23) under the noninformative prior (Table 2; Figure 2). Across all models, the posterior mean RRs were less than 1, suggesting a mortality benefit with TXA. Our estimates were robust across sensitivity analyses incorporating confounding variables, site clustering, and alternative prior evidence. For all parameters, Rhat values were 1.00 and trace plots showed good chain mixing, consistent with adequate model convergence (eFigure in Supplement 1).

**Table 2.** Bayesian Posterior Estimates for Each Model Under Different Prior Assumptions^a^

Model	RR (95% CrI)
CRASH-2	0.91 (0.85-0.97)
CRASH-2 with confounders	0.91 (0.85-0.97)
CRASH-2 deflated^b^	0.91 (0.82-1.00)
CRASH-2 least informative^c^	0.90 (0.75-1.07)
PATCH-Trauma	0.80 (0.65-0.97)
Noninformative	0.82 (0.52-1.23)

Abbreviations: CrI, credible interval; CRASH-2, Clinical Randomization of an Antifibrinolytic in Significant Hemorrhage 2; PATCH-Trauma, Prehospital Antifibrinolytics for Traumatic Coagulopathy and Hemorrhage; RR, risk ratio.

^a^ Robustness to prior specification supports the validity of bayesian findings.

^b^ Refers to an SD of 0.05.

^c^ Refers to an SD of 0.10.

The center and scale of the posterior distribution differed based on the prior specified (Figure 2). More narrow, peaked distributions (CRASH-2, CRASH-2 adjusted, and CRASH-2 deflated models) showed greater certainty. In contrast, the more wide, flat posterior for the noninformative prior model reflected greater uncertainty. Adjusting for known confounders did not have a large influence on the posterior distribution (CRASH-2 adjusted model). Deflating the role of CRASH-2 population size by increasing the SD to 0.05 (CRASH-2 deflated model) broadened the distribution. Increasing the SD to 0.10, further deflating the impact of the CRASH-2 population size, resulted in a wider posterior distribution associated with a lower probability of benefit (CRASH-2 deflated [skeptical] model). Although the point estimates for the CRASH-2, CRASH-2 adjusted, CRASH-2 deflated, and CRASH-2 deflated (skeptical) models were similar, the posteriors with more informative priors yielded tighter distributions. The PATCH-Trauma prior model produced a flatter posterior centered toward the left, corresponding to a higher probability of benefit across risk thresholds. The noninformative prior model yielded the widest distribution and the lowest probability of benefit. Importantly, even assuming a noninformative prior, there was a high probability of TXA benefit (84%) driven by the STAAMP data.

We examined the posterior probability that the RR was below several clinically relevant thresholds (RR <1.0, RR <0.9, and RR <0.8). An RR of 1.0 corresponded to no difference between prehospital TXA and standard care groups. An RR of less than 1.0 corresponded to any mortality reduction with TXA, whereas an RR of less than 0.9 and less than 0.8 represented progressively larger relative risk reductions of approximately 10% and 20%, respectively. These thresholds revealed not only whether TXA was likely to provide any benefit but also the probability that the benefit would reach magnitudes considered clinically meaningful. The posterior probability that TXA reduced 30-day mortality (RR <1.0) was consistently high and ranged from 84% under the noninformative prior to 99% under the CRASH-2 and PATCH-Trauma priors, indicating strong evidence that TXA is associated with reduced 30-day mortality (Figure 2; Table 3). The probability of achieving larger effect sizes (RR <0.9 or RR <0.8) varied across priors. At more stringent thresholds (RR <0.9 and RR <0.8), the posterior probabilities ranged from 39% to 90% and from 0% to 53%, respectively, depending on prior assumption (Table 3).

**Table 3.** Posterior Probabilities and Bayes Factors for RR Thresholds Across Models^a^

Metric	RR <1.0	RR <0.9	RR <0.8
**CRASH-2 prior**			
Probability, %	99	39	0
Bayes factor	>150	0.6	0
Level of evidence	Decisive	No evidence	No evidence
**CRASH-2 prior with confounders**			
Probability, %	99	42	0
Bayes factor	>150	0.7	0
Level of evidence	Decisive	No evidence	No evidence
**CRASH-2 prior (deflated)** ^b^			
Probability, %	98	46	1
Bayes factor	46.6	0.9	<0.1
Level of evidence	Very strong	No evidence	No evidence
**CRASH-2 prior (least informative)** ^c^			
Probability, %	89	55	12
Bayes factor	8.2	1.2	0.1
Level of evidence	Substantial	Anecdotal	No evidence
**PATCH-Trauma prior**			
Probability, %	99	90	53
Bayes factor	87.9	8.6	1.1
Level of evidence	Very strong	Substantial	Anecdotal
**Noninformative prior**			
Probability, %	84	69	48
Bayes factor	5.1	2.3	0.9
Level of evidence	Substantial	Anecdotal	No evidence

Abbreviations: CRASH-2, Clinical Randomization of an Antifibrinolytic in Significant Hemorrhage 2; PATCH-Trauma, Prehospital Antifibrinolytics for Traumatic Coagulopathy and Hemorrhage; RR, risk ratio.

^a^ Probabilities represent the posterior likelihood that the RR is less than 1.0, 0.9, or 0.8, indicating potential mortality reduction with tranexamic acid. Bayes factors quantify the evidence in favor of the hypothesis that RR is below each threshold, with levels of evidence interpreted using the Jeffreys scale.

^b^ Refers to an SD of 0.05.

^c^ Refers to an SD of 0.10.

The BFs provided further support for the probability of benefit. Evidence in favor of a mortality benefit was greatest for the CRASH-2 prior with confounders (BF >150) and PATCH-Trauma prior (BF = 87.9), corresponding to very strong and strong evidence, respectively, and remained positive even under less informative and noninformative priors (Table 3). While the frequentist analysis failed to show a statistically significant difference in mortality sufficient to reject the null hypothesis, our findings suggested a high probability of benefit from prehospital TXA across a range of modeling assumptions and thresholds.

## Discussion

This quality improvement study using a post hoc bayesian analysis of the STAAMP trial found that the probability that TXA would reduce 30-day mortality in patients with trauma at risk for hemorrhage ranged from 84% to 99%, suggesting a TXA benefit, even when assuming a noninformative prior. These findings were robust when accounting for confounders, site clustering, and prior trial evidence. While the original STAAMP frequentist analysis did not detect a statistically significant survival benefit, our bayesian models suggested a high probability that prehospital TXA is associated with reduced mortality. This study illustrates several advantages of bayesian methods, particularly when limitations, such as sample size, may obscure the detection of clinically meaningful results using traditional frequentist approaches.

The CRASH-2 trial demonstrated a significant reduction in mortality with TXA in a large, heterogeneous patient cohort, leading to its adoption in trauma care.^30^ The STAAMP trial found a more nuanced TXA effect that was strongest in specific patient subgroups, such as those with severe shock or who received earlier drug administration.^8^ The PATCH-Trauma trial results further supported the use of prehospital TXA in injured patients.^12^ By using prior clinical trial data to inform our models, this analysis provides a refined estimate of TXA’s effect in patients with traumatic injuries, an approach that may better guide clinical practice than frequentist significance testing alone.

This bayesian approach provides probability estimates of treatment effects across risk thresholds and quantifies uncertainty in clinically relevant terms, highlighting an important distinction between bayesian and frequentist approaches. In the original STAAMP frequentist analysis, the *P* value for the primary outcome was .19. Under the null hypothesis of no difference between prehospital TXA and placebo, the probability of obtaining data as extreme is 0.19, which did not meet the prespecified threshold for statistical significance. In our models, the 84% to 99% probability that TXA would reduce 30-day mortality in patients with trauma at risk for hemorrhage, assuming noninformative and enthusiastic priors, respectively, shows how a negative trial under frequentist statistics and a positive trial under bayesian statistics may address different questions.^14,15,17^ Our results also support previous work that suggested that bayesian methods complement frequentist clinical trials by offering quantitative insights even in inconclusive studies, noting that approximately 30% of negative frequentist studies had a moderate probability of benefit assuming a noninformative prior.^15^ Similarly, a post hoc analysis of the Pragmatic Randomized Optimal Platelet and Plasma Ratios trial estimated that a 1:1:1 study transfusion strategy had a 93% and 87% probability of being superior to a 1:1:2 strategy with regard to 24-hour and 30-day mortality, respectively, even though the original frequentist analysis detected no statistically significant outcome differences.^19,20^ Our findings add to this growing body of evidence and support the importance of bayesian approaches in the evaluation of clinical trial data.

Because prior assumptions could influence bayesian inference, we evaluated multiple priors to reflect varying degrees of clinical belief in TXA efficacy. The CRASH-2 prior incorporated strong trial data supporting a mortality benefit, while deflated versions represented increasing skepticism about its applicability and simulated decreases in the CRASH-2 population size. The PATCH-Trauma prior incorporated information from patients with more severe injuries. Informative priors may either reduce or introduce bias depending on how closely they align with the underlying treatment effect. Therefore, we also evaluated neutral or noninformative priors with a relatively small influence on the posterior distribution (ie, allowing STAAMP data to drive inference). Across all priors, TXA was associated with a probable survival benefit. Noninformative priors often yield estimates similar to maximum likelihood estimates, which are asymptotically unbiased but may exhibit bias in smaller samples.^31^ Moreover, BFs estimated using a noninformative prior are biased toward the null.^29^ Therefore, the noninformative prior may bias our results to be more skeptical rather than favoring the alternative hypothesis as our models suggested.

### Limitations

There were several limitations inherent to this work. First, while bayesian models allow for the incorporation of prior knowledge, prior selection is a source of bias. We addressed this by performing sensitivity analyses with varying priors, including a noninformative prior, all of which supported a TXA-associated mortality benefit. Second, our analysis was limited to 30-day mortality and did not assess long-term functional outcomes, which may be relevant for understanding TXA’s full impact. Additionally, baseline characteristics and outcomes differed across the 3 clinical trials evaluated in this analysis, and we treated 28-day and 30-day mortality as similar when informing our priors. Notably, CRASH-2 enrolled a sicker population with higher overall mortality. Nonetheless, our sensitivity analyses that used both neutral and PATCH-Trauma priors produced similar posterior estimates, supporting the robustness of our findings. Finally, while we adjusted for key confounders in our sensitivity analysis, unmeasured variables may exist. Importantly, this study was a post hoc analysis of a large, prospective, randomized clinical trial and was not prespecified; thus, it should be regarded as hypothesis generating. Future work should aim to prespecify bayesian models and priors during the study design to fully leverage the value of this approach.

While the STAAMP trial did not reject the null hypothesis using frequentist methods, our results suggest a high probability that prehospital TXA may improve survival in patients with trauma at risk of hemorrhage. Although our modeled posterior probabilities were consistently high, for low-cost, low-risk interventions, clinical use may be compelling even with a lower probability of benefit under more conservative risk thresholds. Bayesian approaches may enhance inference and inform clinical decision-making in trauma and transfusion medicine research.

## Conclusions

This quality improvement study using a post hoc bayesian analysis of the STAAMP trial found that prehospital TXA was associated with a high probability of reduced 30-day mortality in patients with trauma at risk of hemorrhage, even when assuming a noninformative prior. This approach quantifies the probability distribution of TXA benefit across clinically relevant risk thresholds and incorporates prior trial data to refine effect estimates. Bayesian methods may help inform clinical practice when frequentist results are inconclusive or sample sizes are small. Although our study design was limited, these findings support the use of prehospital TXA in trauma and highlight the value of bayesian methodology.

## Notes

Supplement 1.
eMethods.
**eFigure.** Model Trace Plots
eReferences.

Supplement 2.
Data Sharing Statement
