# Supplementary material for: Bayesian Statistics to Reanalyze Data From the STAAMP Trial
Source: JAMA Netw Open. 2026 Mar 17;9(3):e262112. doi: 10.1001/jamanetworkopen.2026.2112 (PMC13446807; doi:10.1001/jamanetworkopen.2026.2112)
Supplement: Supplementary file 1 [file jamanetwopen-e262112-s001.pdf]

Supplemental Online Content

Gruen DS, Matuk J, Guyette FX, et al. Bayesian statistics to reanalyze data from the STAAMP trial. *JAMA Netw Open*. 2026;9(3):e262112.
doi:10.1001/jamanetworkopen.2026.2112

eMethods
eFigure. Model Trace Plots
eReferences

This supplemental material has been provided by the authors to give readers additional information about their work.

© 2026 Gruen DS et al. *JAMA Network Open*.

eMethods

Sample R code for model specification using the brms package and No-U-turn sampler.[1,2,3]

```
# Model specification using brms
model <- brm(
       formula = outcome ~ intervention,
       family = bernoulli(link = "log"),
       data   = df,
       prior  = c(
              # Prior for the treatment effect
              set_prior("normal(theta, sigma)",
                     class = "b",
                     coef = "intervention"),
              # Prior for the intercept
              set_prior(
                     "normal(0, 2)",
                     class = "Intercept")),
              chains = 4,
              iter   = 2000,
              seed   = 123,)
# Display model summary including posterior estimates
summary(model_crash)
# Generate diagnostic plots including trace plots
plot(model_crash)
```

© 2026 Gruen DS et al. *JAMA Network Open*.

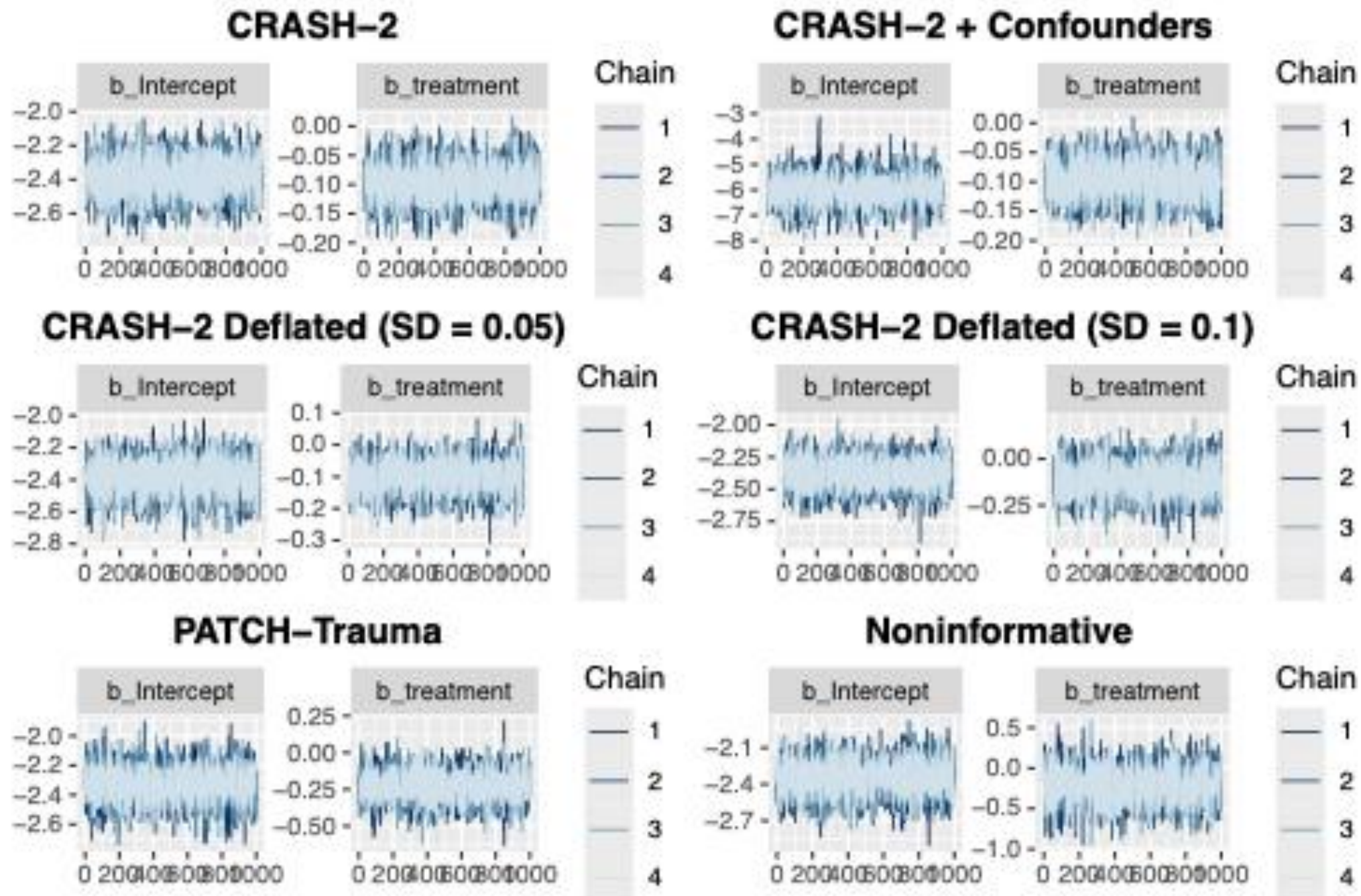

eFigure. Model Trace Plots. Trace plots are displayed for model intercepts and treatment variables.

© 2026 Gruen DS et al. *JAMA Network Open*.

eReferences

1. Bürkner PC. brms: An R Package for Bayesian Multilevel Models Using Stan. Journal of Statistical Software. 2017;80(1):1-28. Available from: http://www.jstatsoft.org/v80/i01/.

2. Bürkner PC. Advanced Bayesian Multilevel Modeling with the R Package brms. The R Journal. 2018;10(1):395. Available from: https://journal.r-project.org/archive/2018/RJ-2018-017/ index.html.

3. Bürkner PC. Bayesian Item Response Modeling in R with brms and Stan. Journal of Statistical Software. 2021;100(5):1-54. Available from: https://www.jstatsoft.org/v100/i05/.

© 2026 Gruen DS et al. *JAMA Network Open*.
