# Supplementary material for: Bayesian Statistics to Reanalyze Data From the STAAMP Trial
Source: JAMA Netw Open. 2026 Mar 17;9(3):e262112. doi: 10.1001/jamanetworkopen.2026.2112 (PMC13446807; doi:10.1001/jamanetworkopen.2026.2112)
Supplement: Supplementary file 2 [file jamanetwopen-e262112-s002.pdf]

Data Sharing Statement

Data
Data available: No
